# On the Injurious Effects of Mineral Poisons in the Practice of Medicine, &c. &c.

**Published:** 1846-10

**Authors:** 


### Art. XIV.

*On the Injurious Effects of Mineral Poisons in the Practice of Medicine, &c. &c.* By Horatio Prater, M.D. PH.D. &c. 12mo, *London*, 1846.

If for once in our lives our gravity might be pardoned for perpetrating a manifest and facile pun, we would say that the work before us is clearly the production of a *Prater*,—an arrant prater of other men's nonsense and his own. It professes to contain an epitome of and commentary on the dietetics of Cornaro, and on the new system of medicine of Raspail, "embracing all the details necessary," as Dr. Prater tells us, "for persons to give this system a fair trial," &c. &c. It is a shrewd compound of twaddle and stuff, intended to be very attractive to noodles and old women, whether in petticoats or breeches. There are some good jokes in the thing, however,—not jokes by the author, who is a dull fellow enough, but statements which his amazing stolidity and ignorance render ridiculous; statements which would figure well in a farce if delivered seriously and in character.

Our author's system of cure participates in the merits of all popular systems, without any of their imperfections. Morison and Mesmer, Priessnitz and Hahnemann, Dickson and Raspail, are all plundered of their finest plumes. Dr. Prater, for example, would give, according to his system, those substances only as medicines which are found as component parts of the body. He would use them separately, but in the very small proportions in which they exist in the human body; and this, Dr. Prater observes, makes his system very similar to the homœopathic. He would also increase or encourage the first symptoms of diseases by medicines which induce similar symptoms, especially when the alimentary canal is to be thoroughly cleared out. "But after this is done, we would not continue (as we presume the regular homœopathist would) the use of medicines tending to keep up such actions, but rather the contrary, since, as we have already stated, such symptoms may often keep up, from the effect of a bad habit (so to speak) the vital principle has got into." (p. 14.)

Dr. Prater has an ingenious proposition respecting keeping up "connexions by blood." He thinks the old folk should have transfusion practised from the veins of their offspring. "If there be anything," he says, "approaching the character of an *elixir vitæ* in nature, it seems more reasonable to look for it in the blood of the adult and healthy offspring than perhaps to any other source." He would also give "oxygen water and solution of deutoxyd of hydrogen." But further—"Eggs and oysters, as evidently *containing a vital principle*, (the italics are not ours,) may be used in such cases, for from their exciting nature they seem, perhaps, to impart a certain quantity of this to those who use them as food." Nay, he holds more recondite views than these, and thinks other means worthy of trial. "Whether, on similar principles, there can be a communication of nervous influence can only be learned by experiment. Does the optic or frontal nerve of an animal *just killed*, laid on the upper eyelid, benefit amaurosis? or the spinal marrow of the same laid or rubbed on the spine of the debilitated old man give any additional strength?" (p. 19.) Aye, answer this, ye who can! We certainly cannot, though we doubt not our great philosopher could if he chose.

Raspail is a sort of monomaniac who has given himself up to the influence of the idea that nine tenths of all diseases are dependent on parasitical animals or worms; an idea, we believe, not unusual amongst medical monomaniacs. The ova of these creatures can penetrate every part of the body; for example, those of the ascarides may enter the lungs with the air at each inspiration. Ascarides never lay their eggs in fæces, "except" (poor things!) "in cases of despair." Acari constitute another very insinuating class of parasites. They will get into flour and cheese, revel in the cracks of wooden pitchers, &c.

"They live in the joinings of *old* furniture, and in houses old or dirty, old wax, collections of dried plants, or animals, in ill-conditioned ulcers, and wherever the *fermentation caséique* can develope itself. Hence it would appear right that the joinings of *all old bedsteads* should be well examined not only on account of bugs, but also of these acari; and, no doubt, it is chiefly to prevent the entrance of these into the mouth or nostrils that Raspail uses his cigarette of camphor *during the night,* when, as we have observed, they generally begin to move about." (p. 41.)

This theory will inevitably recall to the mind of every one who read medical books so long ago as the year 1810, the history of the man in Haslam's most amusing *Illustrations of Madness,* who always slept with his head in a tin saucepan, "to prevent the intrusion of the sprites."

Enraptured by the vermicular theory, our author has given vent to his feelings in immortal verse, and has printed a portion of his poesy. We subjoin a sample for the delectation of our readers:

"We have heard of embalming after man's death,
When for ever has left him the vital breath.
Thus the dead man is kept, no doubt, from decay,
And all sorts of vermin chased from him away:
But here, in this book of Raspail, you will read
That *during our life* of embalming we've need;
That insects assail us within and without;
That from these come all ills—both fevers and gout!
That CAMPHOR and ALOES these only can save us,
And keep that life sound which great Nature gave us.

. . . . . . .

We know some love smoking—smoke half the day;
To all our great smokers here then we say—
Adopt this new plan, smoke, too, *all the night*
Your camphor cigar, which ne'er wanteth a light!
Then by you, *in your life,* no worms will be fed—
Quite enough to be eaten by them *when you're dead!*"—(p. 49.)

Dr. Prater thinks that a deficiency in the quantity of saline matter in the system may be a principal cause of a great many diseases; "in fact, of all those that have an animate origin." He would rather, for his own part, "take a little less aloes and camphor, and a little more saline medicine as *preventives* of worms, than what Raspail recommends." Raspail is good; Prater is better. Nevertheless, Prater likes the notion of ladies smoking camphor cigarrettes in bed; but if this be impracticable, "a piece of camphor inclosed in a linen bag, and placed under the pillow, or tied so as to hang near the mouth and nostrils, would probably be amply sufficient to keep acari and all night vermin from getting into the nose, mouth, or ears, and depositing their ova there." There is one property of camphor, mentioned by Dr. Prater, which might have induced him to caution certain of his readers to take care and make the bag fast, so that it should not slip away and get down into the bed: that property is, "its remarkable power, when sprinkled in powder over the nates, of taking away all venereal appetite, as admitted by many old authors, and by Raspail himself"!

We could fill pages with drolleries like these; things so solemnly writ, and yet so absurd, that the reader would half suspect the whole matter to be a piece of irony, were it not that the asinine stolidity with which it is set forth bears internal evidence of genuineness. And yet there is every now and then bubbling up from his dark depths a bit of brilliant philosophy so very naked as would pass for cunning in other men, but which must convince, as it is intended, every reader that our great Horatio, at least, is a truly modest, simple-minded, truth-loving philosopher of the first water.

For example, he finds in the squabbles of the Royal Society a good reason for glorifying himself. Referring to the clamour lately raised respecting the adjudication of the medals, he observes, "the calm consideration of these unjust proceedings, and a firm conviction of the gross injustice of many writers, and the ignorance of others, have induced me to enumerate my humble labours on the title-page of the present work." And, sure enough, on the title-page, we have appended to the name of our author the following modest enumeration of what he was—even before the publication of the present treatise, videlicet:

"Horatio Prater, M.D., PH.D., Discoverer of the Fusible Compound of Carbon and Silica; of the peculiar Agency of Lime on the Tonicity of Muscular Fibre; of the Permanent Fluidity of the Blood by a Heat of 140°; of the Acceleration of its Coagulation by Ammonia, Carbonate of Soda, &c.; of the *Cause* of the Coagulation of Albumen by Heat; of the Diffusive Power of the Gases; and, conjointly with Fizeau, of Möser's Images; and Author of 'Experimental Inquiries in Chemical Physiology,' &c. &c. &c."

Alas, alas,

"The world knows nothing of its greatest men."

Who, before reading these affecting details, could have imagined that such a genius was lying *perdu* amongst us? And yet who, after reading the great work now before us, can doubt that the ill-used author is much more than is here set forth.

"Horatio, thou art e'en as queer a man
As e'er our observation coped withal."

